# Electrically conductive composite materials with incorporated waste and secondary raw materials

**DOI:** 10.1038/s41598-023-36287-x

**Published:** 2023-06-03

**Authors:** Simon Baranek, Vit Cerny, Rostislav Drochytka, Lenka Meszarosova, Jindrich Melichar

**Affiliations:** Faculty of Civil Engineering, Institute of Technology of Building Materials and Components, Brno University of Technology, Veveří 95, 602 00 Brno, Czech Republic

**Keywords:** Civil engineering, Composites

## Abstract

Silicate composites have very low conductivity in general. It is possible to achieve an electrical resistivity decrease by adding an electro-conductive filler. The conductive mixture consists of cementitious binder, various types of silica sand, and graphite-based conductive fillers. One of the research focusses is partial substitution of ordinary raw materials by alternative components (waste materials by-products and secondary raw materials) and its influence on composite properties. The alternative components studied were fly ash as a partial binder replacement, waste graphite from two different sources and steel shavings as a substitute for conductive filler. Resistivity of cured conductive silicate-based specimens was analysed in relation to changes in physico-mechanical properties in context of microstructural changes in the hardened cementitious matrix (by optical and scanning electron microscopy with energy disperse analysis). Partial substitution of cement by fly ash was found to reduce the electrical resistivity of the composite. Some of the waste graphite fillers significantly reduce the resistivity of the cement composite and increase the compressive strength. It was proven, that is possible to replace primary conductive fillers by secondary raw materials.

## Introduction

Composite materials are one of the most progressive building materials. These materials are used in all branches of industry. Their biggest advantage is the ability to modify properties directly for the given purpose of use. These properties can be modified by using different types and combinations of matrices and fillers^1,2^. The matrix forms the so-called continuous phase of the material and mainly affects the physical and mechanical properties, chemical resistance, thermal conductivity, fire resistance and others of the entire composite material^3^. Matrices are mostly silicate, polymer or geopolymer-based^4^. For most composite materials, fillers significantly reduce the price of the material and further affect bulk density, electrical conductivity, absorbency, etc^5,6^.

An electrically conductive composite can be defined as a composite material that contains a sufficient amount of electrically conductive components to achieve stable and relatively high electrical conductivity. Electrical conductivity is related to resistivity or resistance, it is an inverse value. For solid materials, we can divide electrical conductivity into the internal and surface conductivity. Internal conductivity is related to the structure, amount and nature of the conductive components used, while surface electrical conductivity depends primarily on the water content of the material.

The conductivity of composite materials depends on the mobility of electrons. Cement-based materials usually have resistivity of 6.5**·**10^5^ to 11.4**·**10^5^ Ω·cm^7^, so it can be said that they are not even a good conductor such as copper, which has a resistivity of approximately 1.7**·**10^–8^ Ω·cm^8^, neither good insulator (e.g. Teflon with approximate resistivity 10^15^ to 10^20^ Ω·cm)^9^. By adding conductive components such as soot, graphite, carbon and steel fibers, its resistivity can be significantly reduced while maintaining good mechanical properties^10^.

The key to the excellent electroconductivity of the composite material is the creation of a perfectly electrically conductive network in its structure. The stronger this conductive network is, the greater the electrical conductivity of the material. This is also associated with the maximum force of electric current that can pass through it. Once the interconnected conductive structure is intact in the composite material, the resistance of the material itself is significantly reduced, this limit is called the percolation threshold, and the result is that the subsequent addition of the material no longer affects the resistivity^11^. The electrically conductive network can best be made of materials that are conductive and have an acicular, elongated shape thanks to which they can easily transfer electric current through a non-conductive matrix over longer distances, such as carbon nanotubes, steel fibers, etc. The problem occurs when these acicular components are not in direct contact. therefore, a combination of using several types of conductive elements or increasing their proportion in the material is suitable. Furthermore, the conductivity of the composite is mainly affected by the density, the content of air cavities, the directional orientation of the filler or wires, as well as their dispersion in the composite. Electrical conductivity (principle, internal networks, and structures).

The passage of an electric charge can occur in conductive composite materials through a direct contact line, a tunnel or jump line, and an ion line. The electrons pass through the conductive elements of the material (conductive filler), while the ions come from the matrix mass, which here acts as an electrolyte. Furthermore, electron conduction is effective for steel fibers and carbon fillers such as carbon fibers, carbon nanotubes/nanofibers, and soot^7,12,13^.

The actual electrical conduction mechanism of conductive composite materials is inherently a very complex system. The following types of conductivity coexist in the composite and are interrelated. The relationship between direct current electrical resistance and time may indicate, which type of electrical conductivity (the tunnel line, the jump line and, the ion line) dominates in the composite. When ion conduction is dominant, the DC electrical resistance obviously increases with the measurement time due to the polarization effect; meanwhile, the electrical resistance of the alternating current is constant. In addition, the current–voltage relationship can provide an indication of whether the electrical conductivity of the composite is caused by tunnel/jump or direct contact of adjacent conductive fillers. The relationship between linear current and voltage indicates that direct contact of adjacent conductive fillers is the dominant conductive mechanism. In contrast, the jump conductivity would induce a nonlinear relationship between current and voltage in the electrical conductivity of this composite^1,14^.

All the electroconductive properties of the composite are directly related to the nature and properties of the filler and the matrix and the interactions between them, so it is the easiest way to verify these properties through experiments.

Conductive composites can be divided into two types, conductive fiber composites and composites containing a conductive filler. In practice, however, their combination is most often used to achieve synergistic effects.

Fiber-reinforced conductive composites exhibit improved mechanical properties such as compressive and tensile strength. On the other hand, there can occur areas with reduced conductivity due to low fiber-to-fiber contact, which do not connect the electrically conductive network, creating a "dead-end" between conductive materials such as carbon and steel fibers.

Composites containing conductive fillers show increased conductivity with a resistance value of 10–30 Ω·cm, but have a relatively low compressive strength (less than 25 MPa) which depends on the amount and type of filler. Due to the large specific surface area of these fillers, a higher water content is also required during mixing to compensate for absorption by conductive aggregates such as graphite, soot, or coke. An increase in the water coefficient leads to a significant decrease in compressive strength.

The greatest potential for the use of conductive composites is in civil structures. Thanks to the ability to detect internal stresses, deformations, cracks, and damage, the sensing concrete itself can replace built-in or connected sensors or detectors, which are disadvantageous due to high cost, low service life, limited sensing volume and degradation.

**Figure 1:**
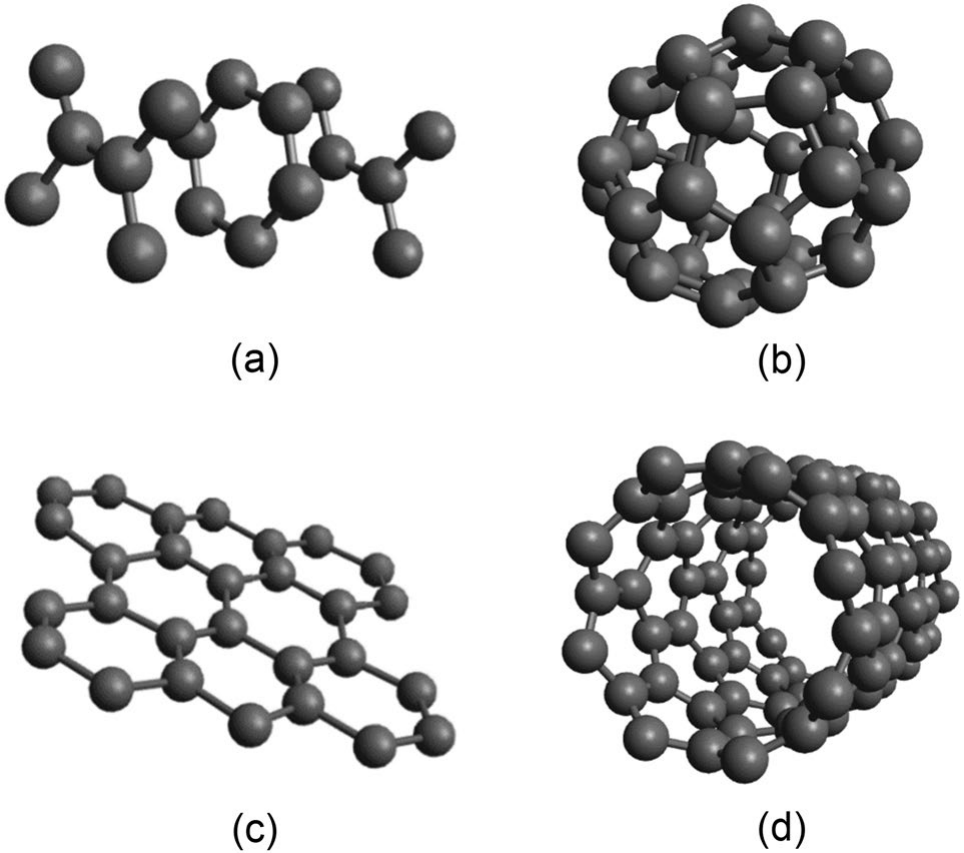
Structure of carbon modification (**a**) graphite; (**b**) fulleren; (**c**) graphene; (d) nanotubes (author's picture).

Fillers with a high carbon content (above 90%), such as soot, are most often used as electrically conductive fillers^15^, carbon dust (graphite)^16^, microsilica^17^, carbon fibres^18^, carbon nano tubes^19^ and nanoparticles such as graphene^20^. The different structure of individual forms of carbon is based on their molecular arrangement. Some layout options can be seen in Fig. 1. The combination of electrically conductive spherical fillers with needle tubes proved to be the most effective. Since these fillers usually significantly reduce the compressive and tensile strength, it is appropriate to further combine them with steel fibers, which also contribute to the reduction of the material resistivity^21^.

Growing environmental and social awareness is forcing the construction industry to place greater emphasis on the environment and the sustainability of developed materials. It also aims to promote the appropriate consumption of natural resources, recycling and reuse of waste materials and raw materials. Secondary raw materials are an important part of the raw material base for most types of industry. Thanks to the use of secondary raw materials, it is possible to reduce the consumption of primary raw materials, which are in most cases non-renewable^22^. Secondary raw materials equally replace and at the same time facilitate technological processes in the production of various materials, thus reducing the energy and material demand of products^23^.

As secondary raw material can be considered any material that have ceased to be waste or are not waste at all. The secondary raw material serves as an input for production and replaces the primary raw material. They usually have the character of by-products of production that have met the conditions and criteria for further use. According to their origin, secondary raw materials can be divided into: by-products, treated waste, materials obtained from products subject to take-back and other end-of-life products, unused input raw materials and materials handed over for new use. Secondary raw materials used in construction include, for example: high-temperature fly ash, silica fume, blast furnace slag, steel slag.

Secondary raw materials in the production of electrically conductive composite materials can be used as a partial replacement of cement, replacement of aggregates, or as a partial replacement of fillers. Secondary raw materials suitable as a replacement for part of the cement, which can also improve the electrical properties of the composite is high-temperature fly ash. The properties and composition of fly ash is variable and depends on the composition of the coal burned, the technology and the combustion process. Due to its composition, fly ash is considered a technogenic pozzolan and contributes to the long-term strengths of the cement matrix and, thanks to the residual carbon content, also increases the electrically conductive properties^24^. Fly ash further improves resistance to chemically aggressive environments, resistance to pressurized water and reduces the development of heat of hydration for use in massive structures^25^. As a substitute for aggregates, it is suitable to use materials with the appropriate fraction and preferably with similar mechanical properties. Raw materials with a high carbon content are particularly suitable as a partial replacement of the conductive filler. For example, waste graphite, which is formed during the production of primary graphite, is suitable for replacing the electrically conductive filler. Furthermore, conductive raw materials based on iron, copper or other conductive materials can be used^26^.

In terms of environmental sustainability, raw materials such as cement and graphite powder are a significant issue and also contribute significantly to the cost of the composite itself. Most studies and research projects mainly use primary carbon powders or mixtures of different fractions. The aim of this study is to examine the potential benefits of using waste and alternative raw materials for electrically conductive composites. To determine their compatibility with the matrix, to specify the benefits but also the disadvantages of using these raw materials.

## Materials

The composition of the electro-conductive composite mixture is based on a verified reference mixture from previous research. Cement (CEM I 42.5 R) is used as a binder. The filler was composed of a mixture of siliceous sands and micro-ground limestone. Graphite powders of a different nature were used as a conductive filler, which were subsequently partially replaced by waste graphite and sawdust. Furthermore, a superplasticizer was used to achieve a suitable consistency^27^.

### Silicate binder

Local Portland cement CEM I 42.5 R (according to EN 197-1^28^) was chosen. The main properties are shown in Table 1.

**Table 1:** Comparison of properties of used materials.

Type of material	Raw material	Resistivity [Ω·cm]	Water absorption [WA5%]	Particle size [mm]	Volumetric weight [kg/m^3^]
Basic materials	Cement	9.12·10^5^	*	0.0–0.25	3140
	Limestone	3.89·10^6^	22	0.0–0.4	2660
	Silica sand 30/31	*	4	0.3–1.0	2630
	Silica sand 35	*	11	0.1–0.3	3380
	Silica sand 1,6–4	*	3	1.4–4.0	3120
	Fly Ash (AM)	2.12**·**10^4^	51	0.0–0.13	1980
Primary graphite	PG-C	0.106	26	0.10–0.65	2250
	PG-F	0.168	190	3.5**·**10^–3^-5.0**·**10^–3^	2410
Waste graphite	WG-GF	0.090	105	3.0**·**10^–3^ -0.65	2460
	WG-HF	0.062	91	3.0**·**10^–3^ -0.65	2320
Waste	Steel shavings	0.022	10	0.5–6.0	3350

*Cannot be measured.

### Silica sands

Aggregate according to EN 12620^29^—aggregate suitable for concrete. It is a mixture of three types of silica sands with fraction of 0.1–4.0 mm. The basic properties of the sands are shown in Table 1.

### Limestone

Micro-ground limestone VBS 40 (commercial name of the product). Limestone was used as a fine filler to modify the consistency of the mixture. The basic properties of limestone are listed in Table 1.

### Admixture: plasticizer

Water-reducing superplasticizer "Stachement 2180.1" in liquid form, based on polycarboxylate according to the EN 934-1 standard^30^.

### Fly ash

It is coal ash, which was produced at a temperature of 1200–1700 °C in the heating plant. Fly ash Arcelor-Mittal (AM) was chosen based on the higher annealing loss (22%) compared to conventional concrete fly ash, which has a maximum loss on ignition of 5% (according to EN 450–1: Fly ash for concrete—Part 1: Definition, specifications and conformity criteria^31^). Increased unburned content in fly ash can potentially reduce electrical resistivity. Higher loss on ignition may also adversely affect the durability of the composite, a factor that will be considered in future research. For comparison, the resistivity of fly ashes with an annealing loss of less than 5% was determined to be in the range of 6**·**10^5^ to 2.2**·**10^6^ Ω·cm, while AM fly ashes reach a resistivity of 2.1**·**10^4^ Ω·cm at 22.2% annealing loss.

Fly ash is generally composed of crystalline and amorphous phases and consists of spherical particles. Fly ash itself has no hydraulic properties. However, when mixed with calcium hydroxide, it reacts to form products similar to those formed when Portland cement is hydrated. Fly ash was selected because of its high annealing loss and potential effect on improving conductivity properties^32^. The key properties of fly ash are stated in Table 1, additional properties in Table 2.

**Table 2:** Selected properties of fly ash (AM).

Selected properties	Unit	Fly ash
Compacted bulk density	[kg/m^3^]	800
Specific surface	[cm^2^/g]	3900
Grain size	[mm]	< 0,125
Loss on ignition	[wt.%]	22.2
Content of active SiO_2_	[wt.%]	36

### Conductive fillers: graphites

Graphite powders with a high carbon content, which were also used in previous research, were used as primary fillers. The graphite fillers used have different properties, grain size, shape, absorbency, etc. Selected properties of the graphite fillers are shown in Tables 1 and 3^27^.

**Table 3:** The main properties of used conductive fillers—graphite powders.

Selected parameters	Unit	Primary graphite PG-F	Primary graphite PG-C	Waste graphite WG-GF	Waste graphite WG-HF
Particle type	[-]	Irregular with rough surface	Flat flakes	Particles of different types	Particles of different types
Bulk density of compacted aggregate	[kg/m^3^]	230	550	540	540
Volumetric weight	[kg/m^3^]	2410	2250	2460	2320
Granularity D (0.1)	[µm]	1.50	83.12	6.70	7.72
D (0.5)	[µm]	2.91	197.02	23.22	36.05
D (0.9)	[µm]	5.26	390.43	117.41	175.38
Specific surface	[cm^2^/g]	19,595	1507	5571	6661
Water absorption capacity WA(5)	[%]	190	26	105	91
Resistivity	[Ω·cm]	0.168	0.106	0.090	0.062

#### Primary coarse graphite PG-C

PG-C graphite powder has flake-type grains. This graphite is natural with a carbon content of 99.5%. The grain size is up to 0.4 mm. The parameters of this graphite are in Tables 1 and 3.

#### Primary fine graphite PG-F

Graphite powder PG-F is natural graphite with a very fine grain. According to the manufacturer, this graphite has improved electro-conductive properties thanks to a modified surface by nanoparticles, see Fig. 3b. This graphite is composed of 99.5% carbon. The particle size is up to 6 μm. The main parameters of this graphite are in Tables 1 and 3.

### Alternative conductive fillers

#### Waste graphite WG-GF

This type of waste graphite powder is a mixture of fine graphite powders. This is the material from the dedusting of the equipment, where various lubricant mixtures are combined and some of the components are sucked out of the equipment in the form of powder (especially graphite) so that this powder does not pollute the working area of the equipment operator. It is mainly fine graphite (up to an average particle size of about 10 microns), with the possibility of a minimum amount of other powders, such as particles of cellulose, water glass, soda can be expected.

#### Waste graphite WG-HF

This is the waste graphite powder that is vacuumed up from the floors of the production halls where the aforementioned primary graphite fillers are produced. It therefore consists of several types of graphite and there is also the possibility of pollution by dust or other foreign particles (e.g. dust from the street, dirt brought in by employees, dirt from forklifts and similar handling equipment, etc.).

#### Steel shavings

These steel shavings are produced as a waste product from the cutting and milling of metallurgical products. These particles are made up of flat, longer fibres which are rolled into spherical particles of 1.0–5.0 mm in size. Steel shavings is a waste from construction and structural steel. Due to their composition and potential electroconductive properties, they were selected as a possible secondary raw material suitable for the formation of electroconductive composites. The disadvantage of this raw material is that a lubricant is used in the production of this material, which must be removed before subsequent processing. The removal of this lubricant will be done with a detergent in water, in which these shavings are washed and then rinsed with clean water just before being added to the mixture.

Properties of waste graphite powders and steel shavings are shown in Tables 1 and 3.

The large specific surface area of graphite powders results in absorption capacity, which subsequently increases the workability of the mixture. As a result, the mixture requires a higher amount of mixing water, which subsequently increases the porosity of the resulting mass, and negatively affects the density of the material, which is related to the lower electrical conductivity of the material.

**Figure 2:**
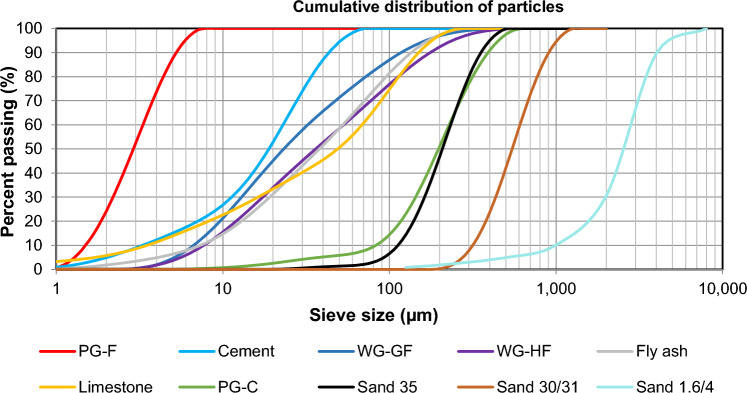
Granulometric curve of used materials.

**Figure 3:**
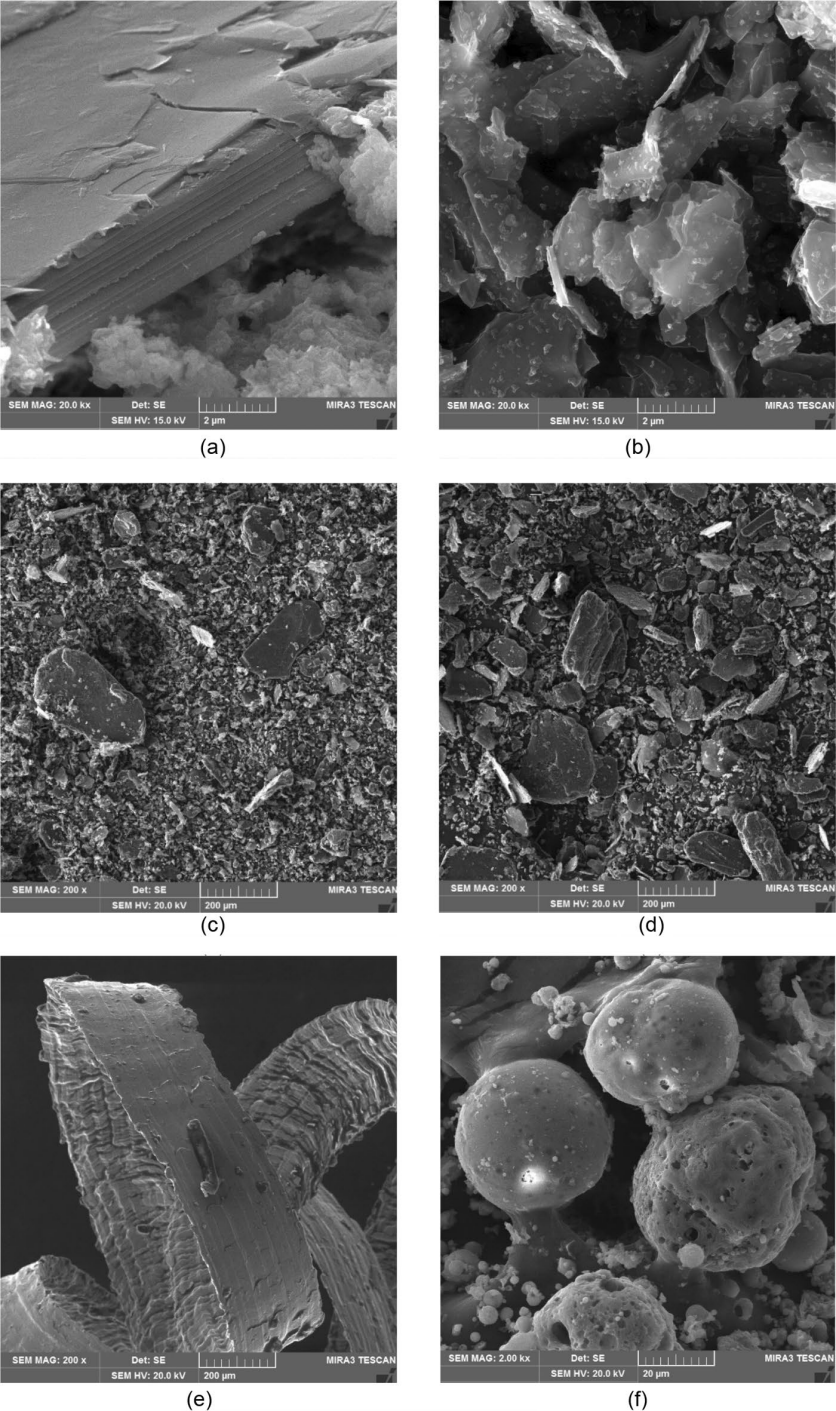
The microstructure of filler particles: (**a**) PG-C particle (**b**) PG-F particle (**c**) WG-GF particle (**d**) WG-HF particle (**e**) Steel shavings, (**f**) Fly Ash AM particle.

Figure 2 shows comparison of grain size S-curves of the materials used. For materials with grains below 1 mm, a Malvern Mastersizer 2000 was used (with a wet dispersion unit; dispersing agent propan-2-ol; dispersion particles were carried out using a sonication unit). For materials with a particle size above 1 mm (Sand 1.6/4), the distribution curve was determined using the sieve method according to the EN 933–1 standard^33^. These materials were also verified in previous research using the same methodology. Input materials are presented in macrophotographs in Fig. 3 (marks distance is 1 mm) and microphotographs in Fig. 3^27^.

As can be seen in Fig. 3 graphite particles are based on clusters of carbon atoms stacked in hexagonal aromatic lamellae. According to the manufacturer, PG-F is declared as graphite with modified electrical conductivity, its surface is modified with nano particles which can be seen in Fig. 3b.

The size, type of particles and their specific surface will affect the consistency of the mixture. The goal is to create a perfectly connected electrically conductive network from these fillers. The better connected this network is, the more stable the current flow. Because of this, fillers with a different type of particles were chosen.

### Developed compositions of materials

#### The cement composites

**Figure 4:**
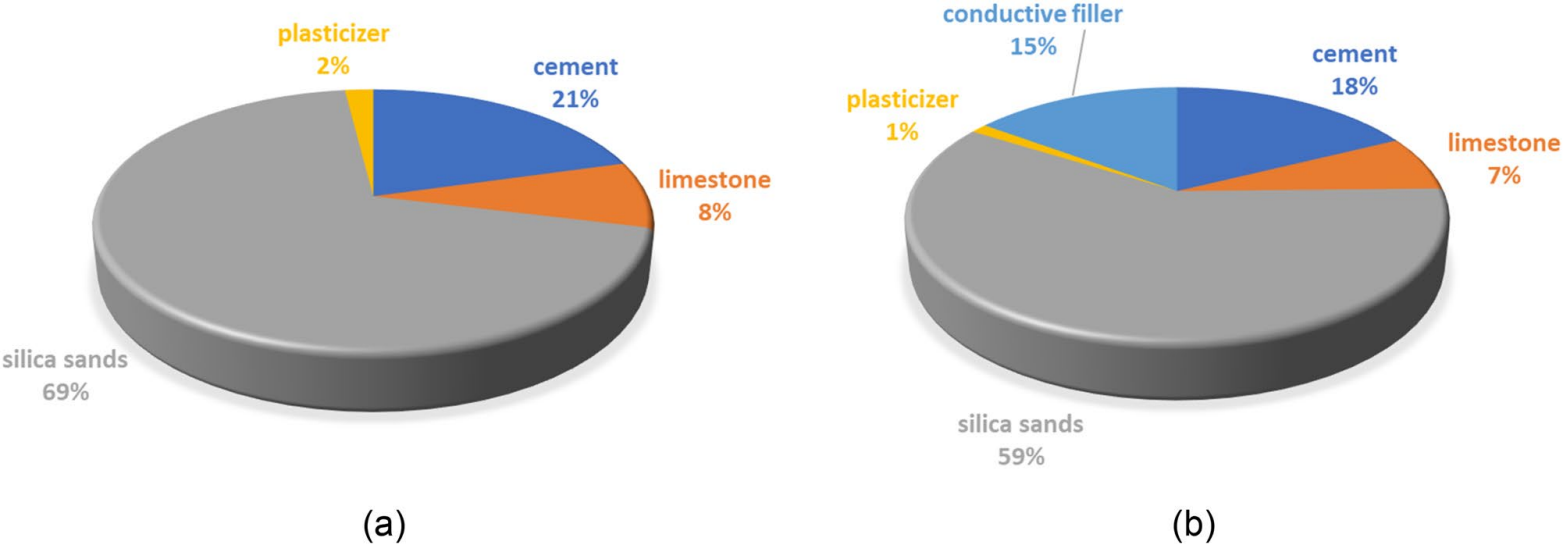
Composition of the mixture for the verification of the resistivity of the composite, amount in weight %: (**a**) basal mixture; (**b**) mixture with the conductive filler.

The composite is based on a silicate base, Portland cement is used as a binder, micro-ground limestone and a mixture of sand with a grain size of up to 4 mm are used as fillers The aggregate curve was compiled according to Fuller. Two primary types of graphite PG-C and PG-F were added as a conductive medium. A plasticizer was added to maintain the appropriate consistency of the mixture. The mixture was moistened to the same consistency of 150 ± 10 mm according to EN 1015-3^34^. Figure 4 shows the composition of the reference mixture and mixtures including the reference conductive composite. Their main properties (with fine and coarse conductive filler) are stated in Table 4.

**Table 4:** Main properties of reference mixtures used for testing basic raw material substitutes.

Parameter:	Unit	Reference composite	Reference composite with PG-C	Reference composite with PG-F
Resistivity saturated	[Ω·m]	11 044	7.5	7.8
Resistivity dried	[Ω·m]	661 693	89.1	4.8
Compressive strength	[MPa]	84.2	4.5	4.5
Flexural strength	[MPa]	15.4	3.3	3.1

The reference composite reaches a strength of 50 MPa after 7 days. This base mix has been designed to be physically and mechanically resistant to ensure suitable mechanical properties even after incorporation of electro-conductive fillers. The aggregate grain size curve was compiled according to Fuller's ideal curves.

#### Mixture with partial cement substitution

Subsequently, the cement CEM I 42.5 R was partially substituted by high-temperature fly ash in the amount of 20%, 30%, and 40% wt.

High-temperature Fly ash AM with a higher annealing loss (over 5%) was chosen as a cement substitute. Fly ash is commonly used as a partial replacement for cement in concrete mixtures. The usual dose for normal use is in the range of 15–35% of the replacement by weight of cement. Detailed composition of mixtures to verify the possibility of partial cement replacement is stated in Table 5.

**Table 5:** Composition of mixtures (in wt. %)—partial substitution of cement by fly ash.

	REF	20%	30%	40%
Cement	17.8	15.0	13.7	12.3
Fly ash	0.0	2.8	4.1	5.5
Limestone	7.1	7.1	7.1	7.1
Silica sands	59.9	59.9	59.9	59.9
Plasticiser	0.12	0.12	0.12	0.12
Conductive fillers	15.0	15.0	15.0	15.0
Composite PG-C w/c ratio	0.51	0.51	0.50	0.50
Composite PG-F w/c ratio	0.72	0.71	0.71	0.70

#### Mixture with partial replacement of primary electroconductive fillers

Waste graphite powders have been proposed as a partial replacement of primary conductive fillers. These are waste graphite WG-GF and WG-HF, these graphite fillers can improve the electroconductive properties, especially by diverse particle distribution, and significantly reduce the consumption of primary conductive fillers, which would have a significant impact on the economic and environmental impact of these materials. Among other alternative substitutes, steel shavings have been proposed, which has also been tested as a substitute for aggregates.

Substitutions of 30% and 50% by volume have been proposed. Both variants will be tested on all three types of replacements and will be tested for the PG-C fine filler composite as well as for the PG-C coarse filler composite, which have been identified as base composites. The composition of the mixtures is given in the Tables 6 and 7.

**Table 6:** Composition of mixtures (in wt. %)—replacement of primary fillers by waste graphite WG-GF and WG-HF.

	REF	30%	50%
Cement	17.8	17.8	17.8
Limestone	7.1	7.1	7.1
Silica sands	59.9	59.9	59.9
Plasticiser	0.12	0.12	0.12
Conductive fillers	15.0	10.0	7.5
Waste graphite	0.0	5.0	7.5
Composite PG-C w/c ratio	0.51	0.49	0.47
Composite PG-F w/c ratio	0.72	0.69	0.68

**Table 7:** Composition of mixtures (in wt. %)—replacement of primary fillers by steel shavings.

	REF	30%	50%
Cement	17.8	17.3	17.1
Limestone	7.1	6.9	6.8
Silica sands	59.9	58.2	57.4
Plasticiser	0.12	0.12	0.12
Conductive fillers	15.0	9.7	7.1
Steel shavings	0.0	7.7	11.4
Composite PG-C w/c ratio	0.51	0.50	0.48
Composite PG-F w/c ratio	0.72	0.63	0.54

## Methods

Based on previous research, the bulk density, specific surface area, grain size and particle size, absorbency and resistivity were determined on the raw materials used. In the case of raw materials, the type of particles was also determined using microscopes. The physical and mechanical properties of the composites were verified, especially the flexural tensile strength after bending and the compressive strength after 28 days. Furthermore, the resistivity of the composites was determined, and finally the structures were analysed using an optical microscope with polarizing filters and a scanning electron microscope.

### Bulk density

The bulk density was established using the standard EN 1097-3^29^.

### Volumetric density

The volumetric density was determined using pycnometric methods in technical alcohol in accordance with the EN 1097-6 standard^31^.

### Specific weight

Specific gravity was determined using a helium AccuPyc II 1340 Pycnometer. This pycnometric method uses inert gases such as helium or nitrogen to determine specific weight.

### Distribution of particles and sieve analysis

For materials with a grain size above 1 mm, the distribution curve was determined using sieve analysis according to the to standard EN 933-1^33^ on a standardized set of sieves.

For materials with a particle size below 1 mm, the distribution curve was compiled using laser diffraction analysis on the device Malvern Mastersizer 2000 according to standard EN ISO 13320^35^.

### Specific surface

The specific surface area of the materials was determined using the BET method according to standard EN ISO 9277^36^.

### Material absorbing power

Due to the high specific surface of electroconductive fillers, their absorbency was determined using a measuring set with a Büchner funnel according to the standard EN 13055^37^. Water absorption is denoted as WA(t), where "t" represents the time, the sample is left saturated. According to previous research, the same time t = 5 min was used in the measurement^27^.

### Resistivity of materials

The resistivity of the input raw materials was determined in the same way as in the previous research, as it is not determined by the standard. Simplicity and repeatability of the measurement were taken into consideration. Measurement cells were produced using 3D printing and subsequently were fitted with electrodes for the measurement of resistivity. Subsequently, the resistivity of the material was determined^27^.

### Measurement process

**Figure 5:**
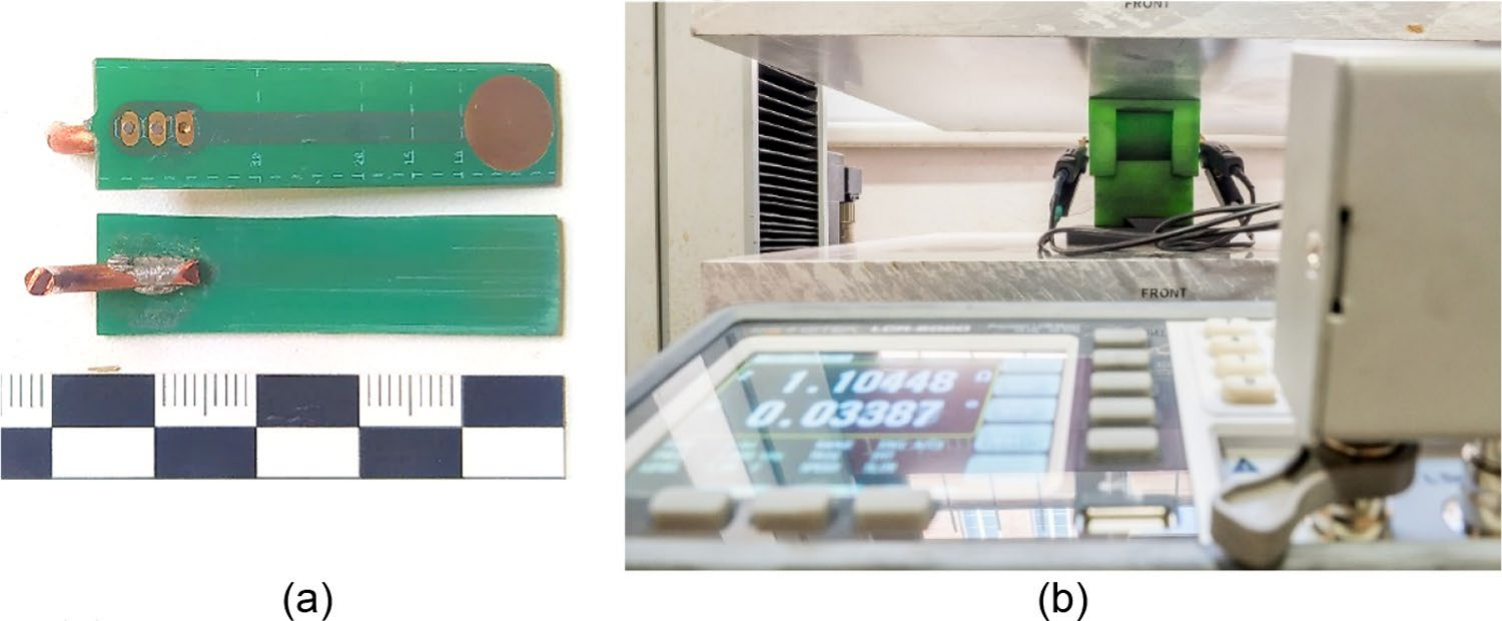
(**a**) Detail of the electrodes; (**b**) The measuring device with a sample during the measurement of resistivity of a graphite powder upon applying pressure of 100 N on the sample.

The prepared device (see Fig. 5) with electrodes was partly filled up to approximately 90% of its capacity with the material. The sample was subsequently compressed using the pressure of 100 N using a press (pressure 1.67 N/mm^2^). Using a table measuring device GW Instek LCR-6020 the impedance of the material was established and subsequently resistivity was calculated from it. The same methodology was also used in previous research^27^.

Based on previous research the device for measuring impedance was created by a 3D printer using a non-conductive plastic PETG. The measuring cell is 10 mm wide, 60 mm long and 50 mm deep. Two opposing electrodes were inserted into the device, 60 mm apart, which are, upon compression of the material subsequently attached to a measuring device (see Fig. 5b). CC.

### Consistency of the fresh mixture

Consistency was determined in accordance with standard EN 1015–3 Part 3: Determination of consistence of fresh mortar (by flow table)^34^. Required spillage was 150 ± 10 mm.

### The manufacturing and storage of test specimens

The materials were dry homogenized for 2 min. After that, water with a plasticizing additive was added. The mixture was then poured into molds 40 × 40 × 160 (mm) according to standard EN 196-1^38^ and densified on the vibration table. Test samples for resistivity determination were fitted with electrodes 12 cm apart in the fresh mixture. For flexural tensile and compressive strength testing, samples without electrodes were created. Subsequently, the samples were placed in a water environment, where they cured for 7–28 days^27^.

### Resistivity of cured specimens

**Figure 6:**
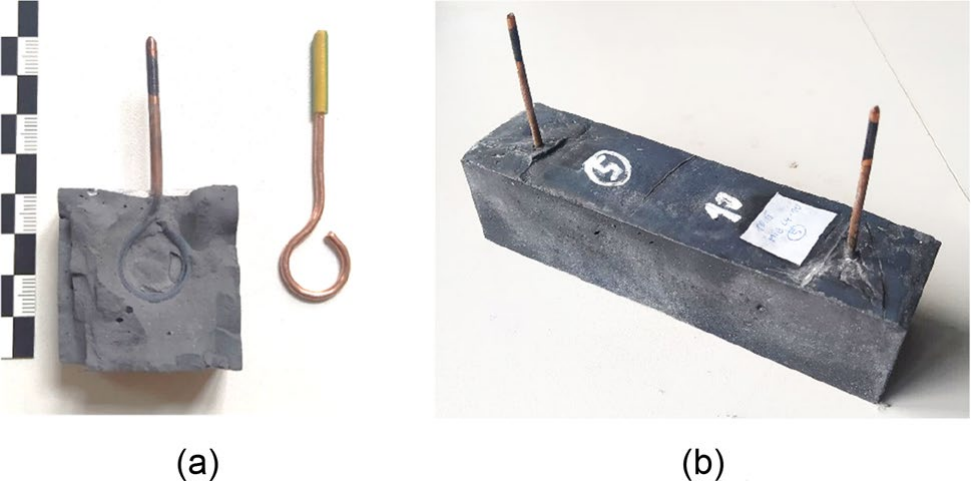
Determination of resistivity: (**a**) a detail of the copper electrode for measuring resistivity in test specimens and verifying full electrode contact with the matrix of composite; (**b**) a test specimen for resistivity measure.

The determination of the resistivity of the test specimens was carried out using methodology following previous research. First, the impedance of the samples was determined and then it was converted to resistivity. During the production of the test samples, copper electrodes (Fig. 6a), located at a distance of 120 mm from each other, were built in, see Fig. 6b. The electrodes were made of copper wire with a diameter of 2.5 mm^27^.

Resistivity was measured after 28 days on samples saturated with water. Subsequently, the samples were slowly dried in a laboratory environment for 90 days. During drying, the samples were weighed, and their resistivity was monitored. Upon reaching a constant weight, the samples were dried at 105 ± 2 °C according to ČSN EN ISO 12570 standard^27^.

The advantages of this resistivity measurement include direct material contact with the electrodes and low risk of material contact loss due to a large number of material changes.

### Porosity

The porosity of the test samples was determined by the relation between the bulk density and specific weight, measured on ultrafine milled samples and using an AccuPyc II 1340 helium pycnometer.

### Physical and mechanical properties

Physico-mechanical properties were determined on test specimens after 28 days of curing. The flexural tensile strength was determined according to EN 12390-5^39^ and compressive strength in accordance with EN 12390-4^27,40^.

### SEM (scanning electron microscope)

The surface of the particles of the materials used and the internal structure of the composites was analyzed using a scanning electron microscope. An electron gun equipped with a cathode with a tungsten filament under a voltage of 15 kV was used. The analysis of the internal structure of cement pastes and composites was performed on fracture surfaces.

### EDX (energy dispersive analysis)

Energy dispersive analysis of characteristic X-ray radiation is a non-destructive method designed to determine the local composition of the material^27^.

### Optical microscopy

The dispersion of filler particles in the cross-section of the material was monitored for the samples, or on the fracture surfaces. A polarizing filter was used to differentiate carbon particles. This filter allows a clear differentiation of the spatially oriented carbon particles in the matrix because they have a different refractive index^27^.

## Results and discussion

### Effect of partial replacement of cement by high-temperature fly ash

#### Effect of partial cement replacement on resistivity of composites

**Figure 7:**
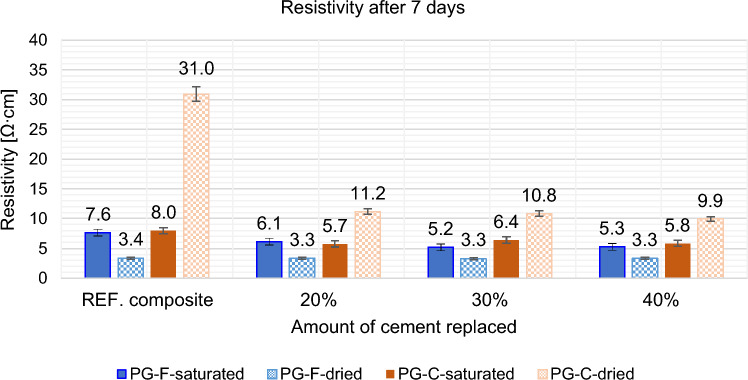
Effect of cement replacement by fly ash on the resistivity of composites after 7 days.

**Figure 8:**
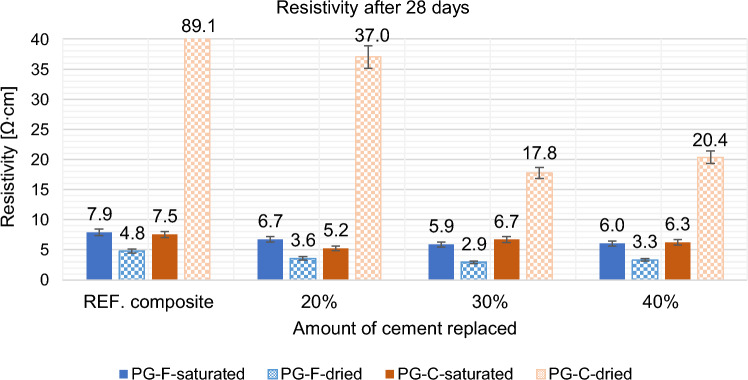
Effect of replacement by fly ash fly ash on the resistivity of composites after 28 days.

A replacement of 20, 30 and 40% of the cement volume was proposed, i.e., approximately 15, 25 and 35% by weight. The effect of partial cement replacement by high-temperature fly ash on the resistivity of the composite was determined after 7 and 28 days in the saturated and dried state. The results are shown in following Figs. 7 and 8.

For the samples with partial replacement of cement by high temperature fly ash, the resistivity of the new composites in the saturated state decreased significantly after 7 days. In the dry state, a significant decrease is only observed for composites containing coarse filler type, see Fig. 7.

When measuring saturated samples, the specific resistance determination is significantly affected by the water content of the sample, but the decrease in specific resistance is still noticeable.

After 28 days of curing, the resistivity of all composites increased, see Fig. 8. Resistivity determined on saturated samples after 28 days copies the trend of resistivity determined after 7 days in the saturated state, resistivity is still affected by the amount of water contained in the sample, in the dried state there are also similar trends compared to resistivity after 7 days.

Based on the above results, it can be said that high temperature fly ash with higher annealing losses is a suitable secondary raw material for reducing the resistance of silicate binder based composites.

**Figure 9:**
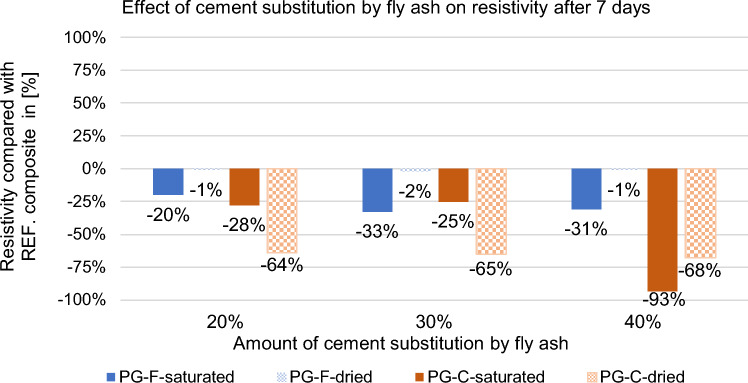
Effect of cement substitution by fly ash on resistivity after 7 days compared to the reference composites (REF).

**Figure 10:**
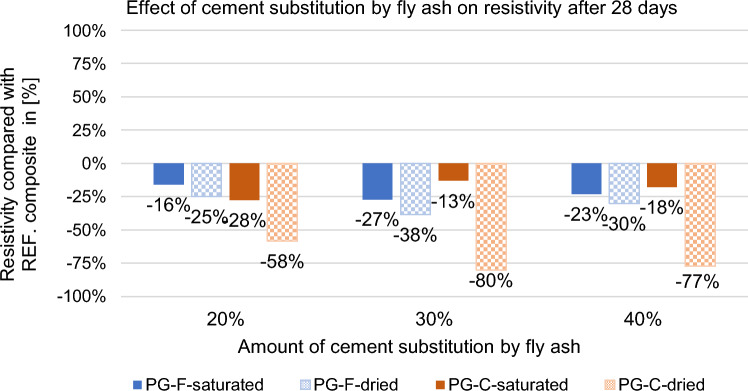
Effect of cement substitution by fly ash on resistivity after 28 days compared to the reference composites (REF).

According to the results given above, the replacement of cement by fly ash is more effective for composites with a coarse type of filler in terms of reducing resistivity, see Figs. 9 and 10. For the composite with fine filler type, the resistance values after 28 days in the dried state decreased by approximately 30% for the composites with coarse filler, this decrease is higher by approximately 60%. As the percentage of cement replacement by fly ash increases up to 30%, the resistances of the samples decrease significantly. The decrease in resistivity is most pronounced for the water-saturated specimens with higher fly ash content. This may be due to the different chemical composition of fly ash and cement binder. Fly ash contains (based on chemical analysis) a higher proportion of quartz than silicon (silicon is a known semiconductor), as well as a small amount of some metals (e.g. chromium, lead, molybdenum, barium) and also inorganic salts in the amount of 1380 ml/l. Inorganic salts dissolve in contact with moisture and form an electrolyte, creating an ideal environment for electrolysis. This finding corresponds to what Philathottathil described in his work^41^. Inorganic salts, when dissolved in water, cleave into positively charged cations and negatively charged anions. This phenomenon is called electrolytic dissociation. The pore structure filled with an electrolyte (an aqueous solution of ionically bound compounds) tends to behave as a better conductor compared to a pore structure filled with water without dissociated salts. After the samples have dried, these resulting electrolytic paths are interrupted, the pores filled with water are released and an empty space is created after them. As a result, the resistivity of the PG-C coarse-grained graphite samples increased significantly due to the lack of proximity to the individual grain surfaces of the conductive filler. On the other hand, in the case of samples with a finer-grained filler, there is a clear trend of decreasing resistivity with drying of the sample. In the case of samples with a fine-grained conductive filler, the conductivity network is so dense that the pore water makes it more difficult for electrons to transfer between the individual grains. The optimal dose of cement replaced by fly ash is 30% in terms of resistivity (both for saturated and dried samples).

#### Effect of partial cement replacement on bulk density and porosity of the material

**Figure 11:**
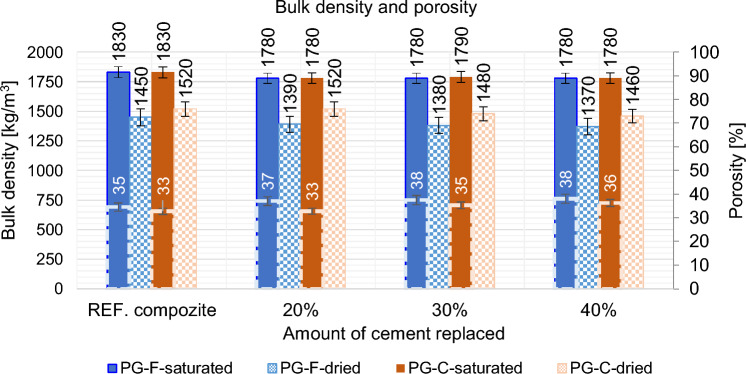
The effect of partial replacement of cement by high temperature fly ash on the bulk density and porosity of the composite.

The partial substitution of cement by high temperature fly ash slightly increases the porosity of the material and consequently reduces the bulk density of the composite. This may be mainly due to the slower hydration of fly ash and the spherical, cavernous character of the particles. The pores are not filled with hydration products to the same extent as in the case of higher cement doses. The porosity increases with increasing dosage of high temperature fly ash see, Fig. 11.

#### Effect of partial cement replacement on mechanical properties of composites

**Figure 12:**
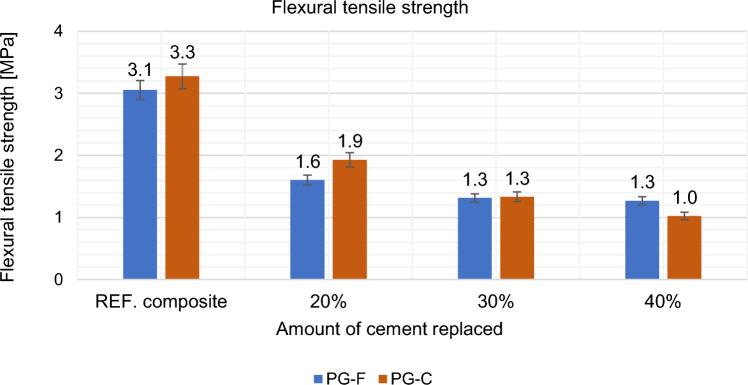
Effect of cement replacement by fly ash on the flexural tensile strength of the composites after 28 days.

**Figure 13:**
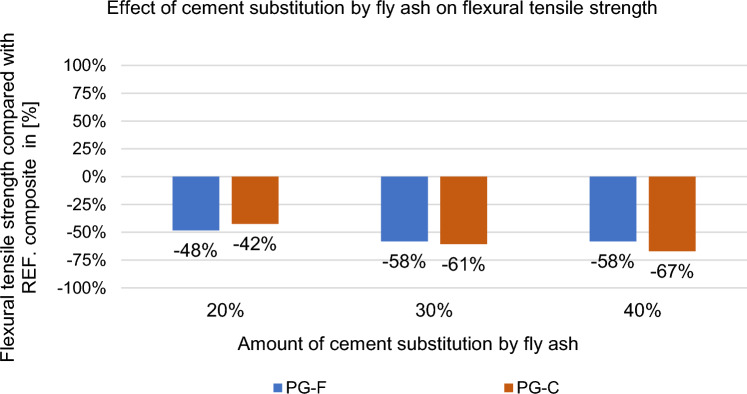
Effect of cement replacement by fly ash on flexural tensile strength of the composites after 28 days compared to the reference composites (REF).

**Figure 14:**
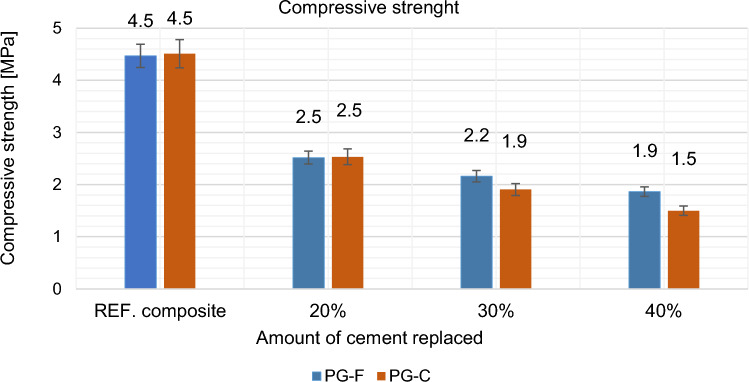
Effect of cement replacement by fly ash on the compressive strength of the composite after 28 days.

**Figure 15:**
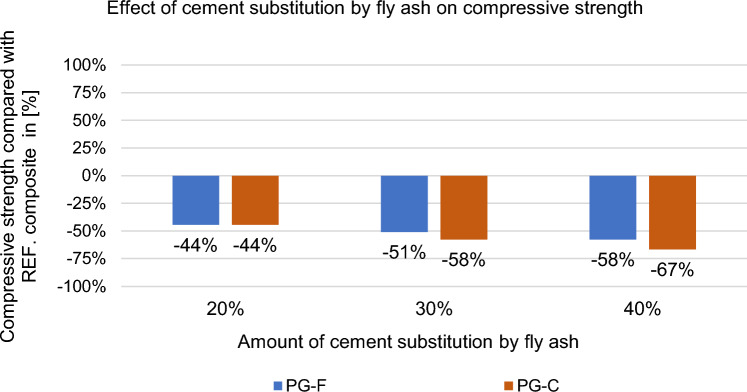
Effect of cement replacement by fly ash on compressive strength of the composites after 28 days compared to the reference composites (REF).

Flexural tensile strength and compressive strength were tested on 40 × 40 × 160 (mm) specimens. These mechanical properties were monitored after 28 days in the saturated state. The results are shown in Figs. 12, 13, 14 and 15.

As can be seen above, flexural tensile strength decreases with increasing partial replacement of cement by high-temperature fly ash, these differences may be caused by a slowed growth in strength caused by fly ash.

Reduction of compressive strength is like the flexural tensile strength affected by slowed increase in strength caused by the replacement of the binder component with fly ash^42^.

Substitution of cement by fly ash decreases the strength after 28 days (approximately 50%), see Figs. 13 and 15. This decrease in strength is due to the slowed increase in strength that is typical for Fly ash. While cement behaves according to the Powers model^43^. According to this theory strength arises from two general kinds of cohesive bonds: physical attraction between solid surfaces and chemical bonds; and is basically related to the colloidial state of the major product of the reactions between Portland cement and water, and to the spatial concentration of this product (cement gel)^43^. Hydrated portland cement with typical clinker mineral content of C_3_S and C_2_S produces about 20 to 25 wt.% Ca(OH)_2_, because of this, cement achieves higher flexural tensile strengths and in compressive strength^44^. Compared to cement, fly ash begins to react with Ca(OH)_2_ later, but significant amounts of Ca(OH)_2_ and fly ash still remain unreacted even after 90 days of hydration^45,46^. The reaction products, mainly (CSH), have a lower ratio of CaO:SiO_2_ (c/s)^45^. The mechanical properties of the composite with the replacement of the binder with fly ash are related to the curing conditions and the curing time, which is slowed down due to the above-mentioned facts. Until the pozzolanic activation of fly ash starts (according to Ref.^47^ it starts approx. after 90 days), the strength of the composite decreases as the amount of the substituted binder increases^48^.

### Effect of partial replacement of primary graphite fillers by waste graphite and steel shavings

#### Effect of primary conductive fillers partial replacement on resistivity

**Figure 16:**
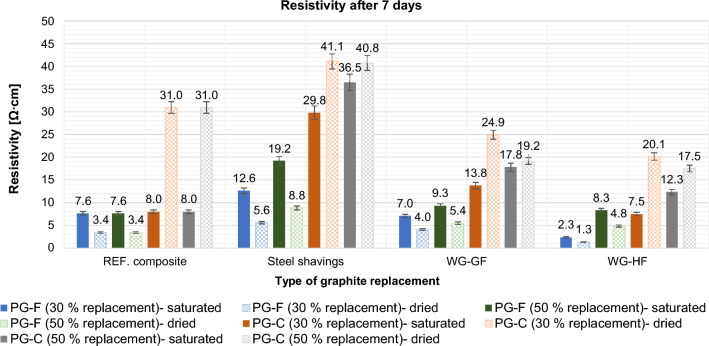
Effect of primary conductive filler replacement on composite resistivity after 7 days.

**Figure 17:**
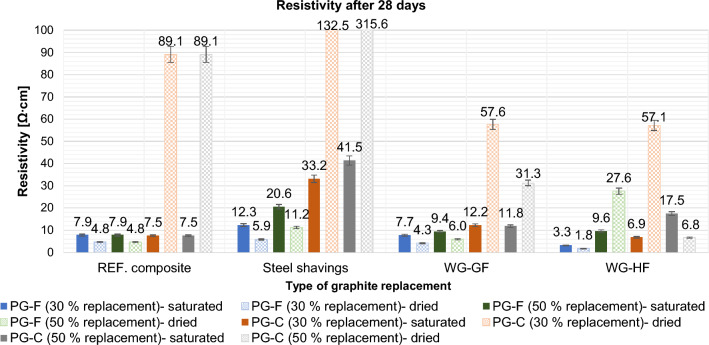
Effect of primary conductive filler replacement on composite resistivity after 28 days.

Resistivity was determined in dry and saturated states after 7 and 28 days. The results are shown in following Figs. 16 and 17.

After 28 days in saturated and dried state due to replacements of 30% and 50% by volume, the resistivity values changed according to the following graph.

**Figure 18:**
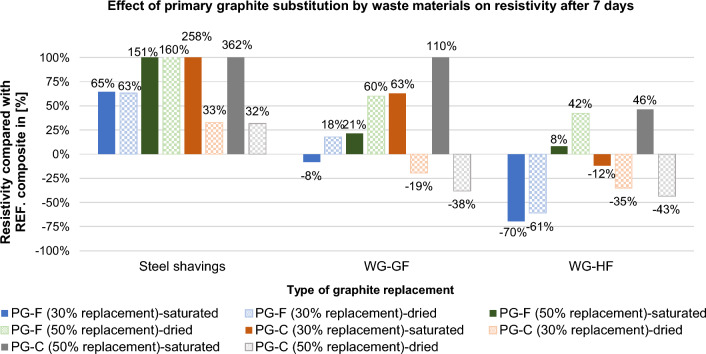
Effect of 30% and 50% primary graphite substitution on the resistivity after 7 days compared to the reference composites (REF).

**Figure 19:**
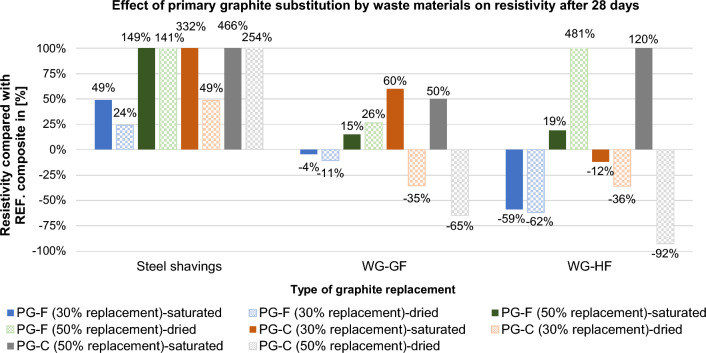
Effect of 30% and 50% primary graphite substitution on the resistivity after 28 days compared to the reference composites (REF).

Of the wastes tested, waste graphite is suitable as a substitute filler. Steel shavings increase the resistivity significantly, especially in the saturated state of the samples, see Figs. 18 and 19.

For a composite with a fine type of filler (PG-F), the most suitable filler replacement is 30% by volume of WG-HF. For composites with a coarse type of filler (PG-C), a 50% replacement by waste graphite WG-HF is most suitable. Steel shavings in the saturated and dried state significantly increase the resistivity. See Figs. 18 and 19, the cause is explained below.

#### Effect of primary conductive fillers partial replacement on bulk density and porosity of the material

**Figure 20:**
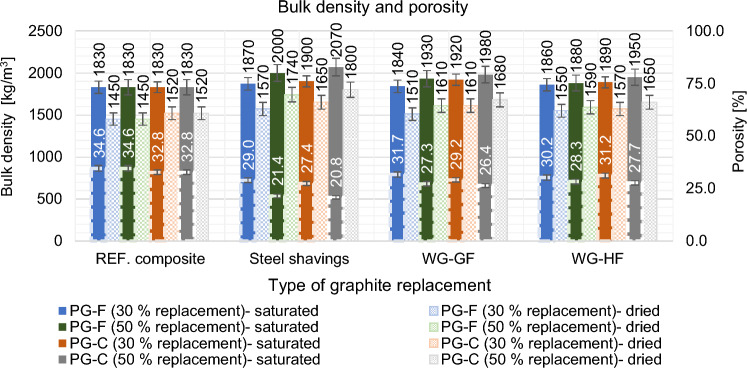
The effect of partial substitution of primary fillers on the bulk density and porosity of the composite.

According the Fig. 20, the addition of steel shavings achieved lower porosity and higher bulk density of the material. This is mainly due to the character of the particles, which have low water absorption and have a lower specific surface area than the primary graphite fillers. Replacing the primary graphite fillers with waste fillers also slightly reduces the porosity, mainly due to the lower absorption of the fillers and the lower specific surface area. This also results in a more compact material that is better able to conduct electrical current.

#### Effect of primary conductive fillers partial replacement on physical–mechanical properties of composites

**Figure 21:**
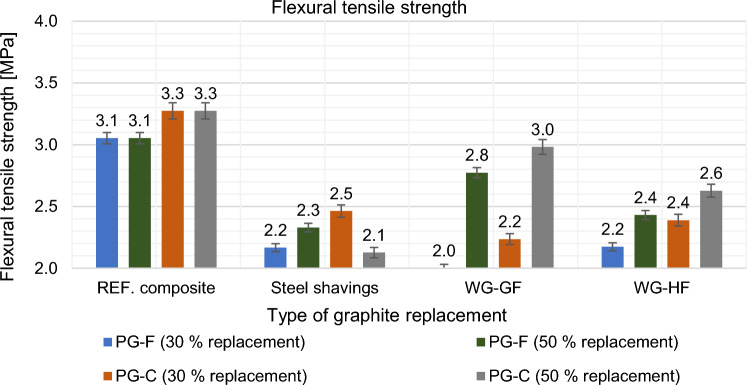
Effect of primary graphite substitution on flexural tensile strength of composite after 28 days.

**Figure 22:**
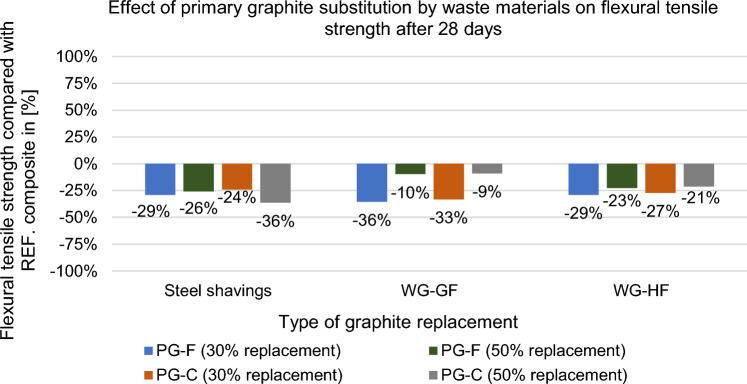
Effect of primary graphite substitution on flexural tensile strength of composite after 28 days compared to the reference composites (REF).

**Figure 23:**
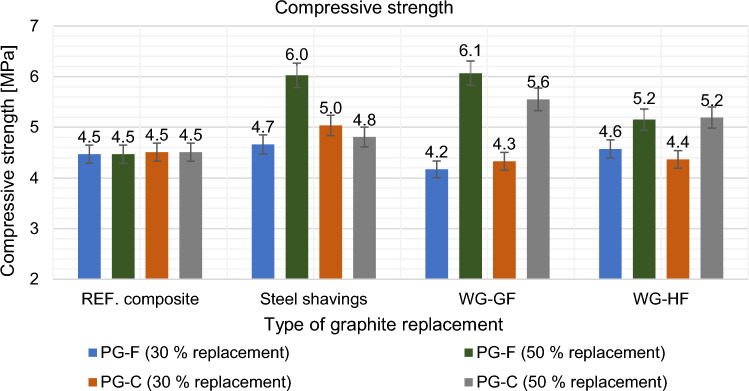
Effect of primary graphite substitution compressive strength after 28 days.

**Figure 24:**
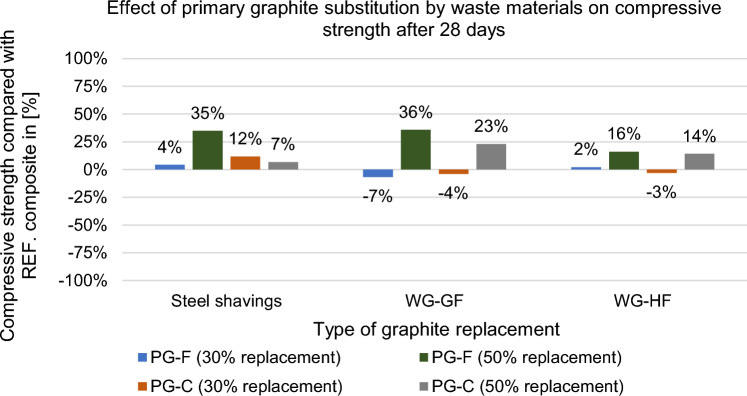
Effect of primary graphite substitution on compressive strength after 28 days compared to the reference composites (REF).

The mechanical properties were tested after 28 days in the saturated state. The results are shown in Figs. 21, 22, 23 and 24.

Based on the results given in the Figs. 21 and 22 above, the optimal variant based on flexural tensile strength for the composite with a fine type of filler is the most suitable replacement by WG-GF in the amount of 50%, for the composite with a coarse type of filler the optimal WG-GF replacement in the amount of 50%. Overall, it can be stated that with the replacement of primary fillers by waste, the flexural tensile strength decreases.

Compressive strength was determined after 28 days and the results are shown in Figs. 23 and 24.

It was found that the replacement of a part of the primary filler with waste raw materials improved the physical and mechanical properties in most cases, which may be due to a more suitable distribution of particles supplementing the grain size curve. For the fine filler type composite, the replacement was not advantageous in comparison of the decrease in resistivity to the decrease in strength.

**Figure 25:**
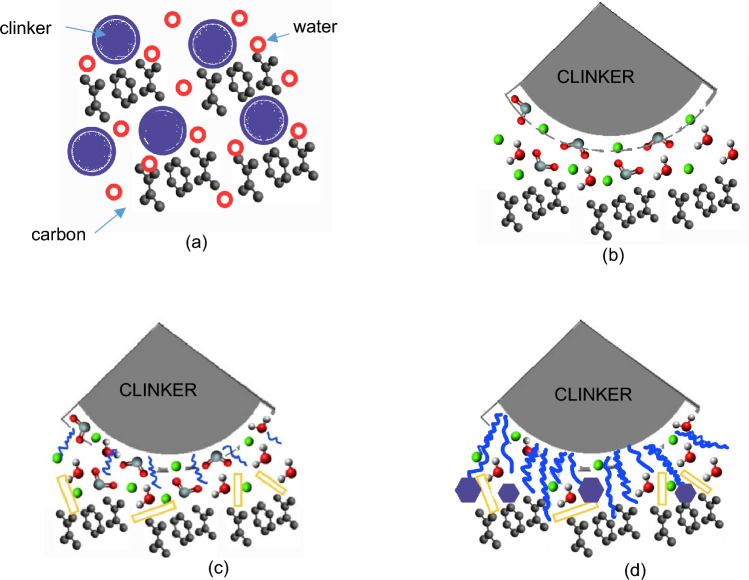
Hydration of the cement matrix with a carbon filler: (**a**) mixing of the input materials; (**b**) the beginning of cement hydration; (**c**) sequential formation of AFt and CSH and CH phases; (**d**) consolidation and stabilization of CSH, CH phases, formation of AFm phases.

Waste graphite fillers, which significantly reduce the resistivity of the composite and increase the compressive strength, have proven to be a useful replacement for primary conductive fillers. WG-HF waste graphite is the most suitable substitute for both types of composite. For the composite with the fine type of filler PG-F, due to the 30% substitution, the resistivity decreased by 60% in all types of measurements compared to the reference mixture from primary raw materials. In the composite with a coarse type of filler, the most effective replacement was 50%, in which the dry resistivity was reduced by up to 92% after 28 days. The only disadvantage of this replacement is the decrease in flexural tensile strength, which is around 20%. The mechanism of graphite incorporation in the cement matrix was described by Wang in his work^49^. Graphite does not contribute to the hydration of portland cement, it can only affect the certain decrease in the degree of hydration of cement and the basicity of calcium hydrosilicates^50^. Theoretical course of hydration of cement particles in the vicinity of carbon particles is shown in Fig. 25.

**Figure 26:**
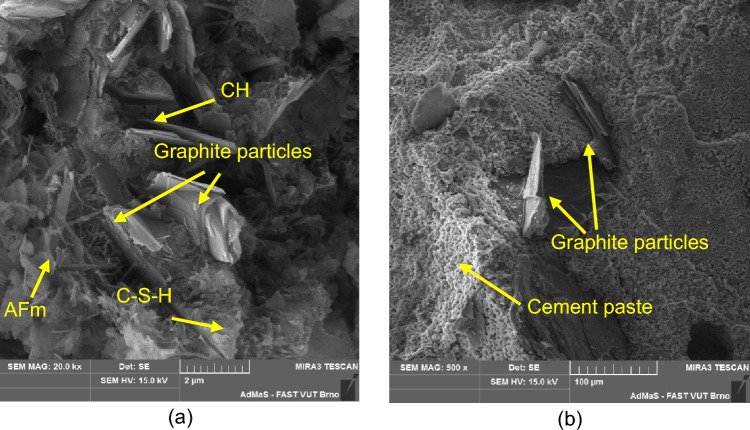
Detailed images of the incorporation and dispersion of the carbon filler in the cement matrix (**a**) PG-F; (**b**) PG-C.

The fact that the graphite filler is inert and does not enter into hydration reactions is clearly evident from the electron microscope images (Fig. 26). The individual single layer platelets clearly come out of the hydrated cement paste that coats them.

**Figure 27:**
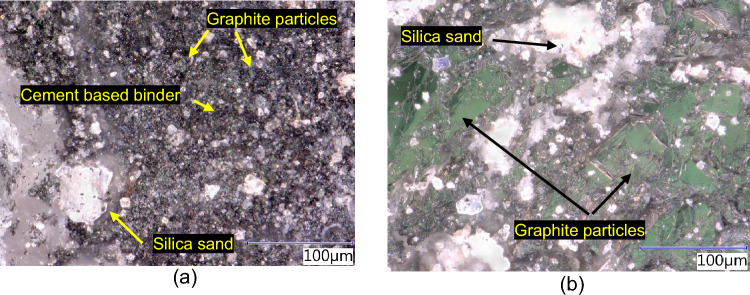
Distribution of individual components of the composite in the structure, (**a**) PG-F; (**b**) PG-C.

The individual components of the binder do not separate the graphite particles perfectly (see Fig. 27), thus creating an opportunity for the formation of power line paths. The filler components are selected to be either conductors or semiconductors in terms of conductivity. Electronic or hole conductivity can be used here. Good permeability of the conductivity paths was demonstrated by reducing the resistivity of the samples by the used graphite-based conductive filler.

**Figure 28:**
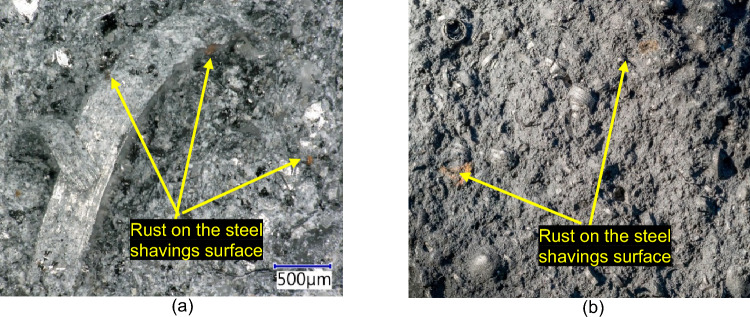
Detailed images of the incorporation and dispersion of steel shavings in the composite: (**a**) detailed image of the steel shavings in the matrix; (**b**) Macroscopic image of scattered shavings in the composite.

The incorporation of fillers based on steel particles brings with it negative phenomena, such as insufficient passivation of the filler leading to their corrosion due to the passing electric current through the sample. (see Fig. 28). Zhang^51^ described how electrochemical corrosion of steel in a cement matrix occurs. The corrosion rate can be controlled by either the anode or cathode partial reaction, or both at the same time. The anode reaction takes place during the oxidation of the metal itself. The cathodic (depolarization) takes place as part of the reduction of the oxidizing component of the solution.

1 $${Fe}^{2+}+2{OH}^{-}\to Fe{\left(OH\right)}_{2}$$

2 $$Fe{\left(OH\right)}_{2}+{O}_{2}\to Fe{\left(OH\right)}_{3}$$

The surface layer of rust on the cathodes makes it difficult to move the electrons and thus increases the resistivity of the mass, which has also been proven in testing the resistivity of samples with steel shavings as a conductive filler.

## Discussion

In this study, it was shown that in order to achieve a suitable electrical conductivity, it is possible to use as conductive fillers not only primary raw materials, but also waste or secondary raw materials.

The use of AM high-temperature fly ash, which is characterized by higher loss on ignition and the proportion of heavy metals and inorganic salts, has led to a significant reduction of resistivity. It has been shown that the use of this type of fly ash, which cannot be used for common building materials, leads to an improvement in the electroconductive properties of the composite at a dosage of 20%. The use of fly ash is also advantageous from an environmental point of view. Its use saves primary raw materials, namely Portland clinker, which contributes to reduction of the carbon footprint. The disadvantage is roughly a 50% reduction of short-term strengths (28 days) compared to reference composites. Fly ash enters hydration reactions differently compared to Portland clinker. Fly ash is known for its pozzolanic and latent hydraulicity. Helps reduce heat of hydration. Amorphous SiO_2_ enters hydration, which reacts with Ca^2+^ ions and water to form the calcium hydrosilicate phase. The increase in strength is significantly slower compared to Portland clinker and it can be assumed that composites with cement replaced by fly ash will reach comparable strengths only at the age of about 90 to 180 days. The study of long-term strengths will be the subject of further research. An adverse effect of the addition of fly ash to the mixture is an increase in the porosity of the composite, which is partly due to slower hydration and in particular the character of the pleosphere-shaped particles. Research has shown that the use of fly ash as a 20% binder component replacement and primary graphite replacement by secondary raw materials is advantageous in terms of reducing the resistivity of composites.

One of the variants of conductive fillers that have been used are steel shavings (waste from metal production created during cutting and grinding of structural steel). These materials have been found to be unsuitable for use in silicate-based matrix composites. The fineness of the shavings in combination with the roughly treated surface of the particles in contact with air humidity leads to very fast electrochemical and/or oxidative corrosion, during which a very thin surface rust is formed. This surface corrosion consequently prevents the transmission of free electrons and electric current.

Waste graphite fillers were tested as a replacement for primary fillers. Waste graphite WG-HF is composed of graphite residues, dust and impurities, which were suspended from the floors of the company's production halls. It is mainly characterized by fineness, lower absorbency and a diverse range of particle sizes. WG-GF waste graphite was also used, which is composed of mixtures of fine graphite powders. It is a material from a dedusting device in which various lubricants are mixed. It has been proven that due to the appropriate particle size distribution, these waste materials achieve more efficient structural interconnection of the electroconductive network compared to the primary graphite powders alone. Waste graphite fillers also improved the physical and mechanical properties of the composites. Furthermore, it has been found that by mixing fine particles into composites with a coarse type of filler, their resistivity in the dry state can be significantly reduced, due to the reduction of the distance of potentially conductive fragments in the mass.

One of the disadvantages of graphite powders is their high specific surface area and absorbency, these properties cause a relatively high increase in the porosity of the material. In general, a more compact material has better electrical properties provided it contains sufficient conductive elements. Waste graphite fillers provide the advantage of significantly lower water absorption than primary graphite powders.

Moisture significantly affects the electrical conductivity of silicate composites. The biggest differences are observed while using a coarse type of filler (after drying) its resistivity increases up to 10 times. The opposite phenomenon was observed in composites with a fine type of filler when the resistivity decreased approximately 2 times when the material was dried. Furthermore, the possibility of filling composites with mixtures of coarse and fine graphite was verified. The fine type of graphite fillers in coarse primary graphite composites significantly reduced the resistivity in the dried state.

According to microstructural analysis was determined coherence between the composite conductivity and the shape and dimensions of used conductive filler. Coarse graphite particles are less able to spread electrical charge because the distance between each particle surface is bigger and the conductive path is often damaged. The distance of these parts is also significantly affected by the degree of hydration of the composite, where it was shown that samples based on coarse graphite achieved significantly higher resistivity in the dry state after 28 days of maturation than samples after 7 days of maturation. In the case of the fine type of graphite, this phenomenon has not been recorded.

## Conclusion

In this study, the possible use of waste alternative materials as electrically conductive fillers for silicate composites was proved. The effect of moisture and hydration of cement on the electrically conductive properties was also investigated. It has been proven that:

- High-temperature fly ash with higher loss on ignition (above 5%) as a binder replacement significantly improves the electrically conductive properties of silicate composites.
- Waste graphite, due to the natural diversity of particle size, can more effectively form a more stable electrically interconnected conductive network in the matrix of the composite, thus improving the electrically conductive properties, also slightly improved the physical–mechanical properties.
- Steel shavings are not suitable as electrically conductive fillers. The fineness of the steel particles, together with the surface roughness and moisture, leads to very rapid electrochemical and/or oxidative corrosion, which forms a rust on the surface of the particles that insulates the particles from electric current.
- Moisture significantly affects the electrically conductive properties of the composites, which can vary up to tenfold.
- The presence of water in composites with coarse graphite decreases the resistivity, whereas water in composites with fine filler increases the resistivity.
- The degree of hydration of the cement affects the electrically conductive properties. For the composite with coarse filler type, the resistivity increased significantly between the measurement at 7 and 28 days. For the composite with fine filler type, this phenomenon was not observed.

Future research may be focused on durability and physical–mechanical properties of materials especially for composites with fly ash content which has a higher annealing loss. Another possible direction of research is in extending and refining the effect of humidity, temperature and their combination on electrically conductive properties.

## Data Availability

The datasets used and/or analysed during the current study available from the corresponding author on reasonable request.
